# Piwi-interacting RNA (piRNA) expression patterns in pearl oyster (*Pinctada fucata*) somatic tissues

**DOI:** 10.1038/s41598-018-36726-0

**Published:** 2019-01-22

**Authors:** Songqian Huang, Yuki Ichikawa, Yoji Igarashi, Kazutoshi Yoshitake, Shigeharu Kinoshita, Fumito Omori, Kaoru Maeyama, Kiyohito Nagai, Shugo Watabe, Shuichi Asakawa

**Affiliations:** 1 Graduate School of Agricultural and Life Sciences, The University of Tokyo, Bunkyo-ku, Tokyo 113-8657 Japan; 2 Mikimoto Pharmaceutical CO., LTD., Kurose 1425, Ise, Mie 516-8581 Japan; 3 Pearl Research Laboratory, K. MIKIMOTO & CO., LTD., Osaki Hazako 923, Hamajima, Shima, Mie 517-0403 Japan; 4 School of Marine Biosciences, Kitasato University, Minami-ku, Sagamihara, Kanagawa 252-0313 Japan

## Abstract

Piwi-interacting RNAs (piRNAs) belong to a recently discovered class of small non-coding RNAs whose best-understood function is repressing transposable element activity. Most piRNA studies have been conducted on model organisms and little is known about piRNA expression and function in mollusks. We performed high-throughput sequencing of small RNAs extracted from the mantle, adductor muscle, gill, and ovary tissues of the pearl oyster, *Pinctada fucata*. RNA species with sequences of approximately 30 nt were widely expressed in all tissues. Uridine at the 5′ terminal and protection from β-elimination at the 3′ terminal suggested that these were putative piRNAs. A total of 18.0 million putative piRNAs were assigned to 2.8 million unique piRNAs, and 35,848 piRNA clusters were identified. Mapping to the reference genome showed that 25% of the unique piRNAs mapped to multiple tandem loci on the scaffold. Expression patterns of the piRNA clusters were similar within the somatic tissues, but differed significantly between the somatic and gonadal tissues. These findings suggest that in pearl oysters piRNAs have important and novel functions beyond those in the germ line.

## Introduction

The pearl oyster (*Pinctada fucata*), an economically and ecologically important oceanic mollusk, is one of the best studied species with respect to the biomineralization processes during pearl and shell formation^1–4^. It is widely distributed across Japan and China, and substantial quantities of pearls are harvested from it^5^. Modern pharmacological studies show that pearl powder are important in human biochemical reactions, which can promote human metabolism and regulate physiological functions^6,7^.

To date, hundreds of pearl formation related genes have been identified^8–12^, but knowledge regarding small RNA function in the pearl oyster is limited. Small RNAs, <200 nt in length, are usually non-coding RNA molecules that play a pivotal role in the regulation of gene expression at the post-transcriptional level^13^, especially for signaling pathways involved in development, cellular differentiation, proliferation, apoptosis, and oncogenesis^14^. Piwi-interacting RNAs (piRNAs), a recently discovered class of small non-coding RNAs, are distinguished from microRNAs (miRNAs) by their slightly longer sequences and germline specific expression^15–18^, which prevents genomic damage caused by transposable element reactivation^19^. Two common features of the piRNAs in all systems analyzed to date are that they are derived from repetitive transposon rich regions of the genome and they have 2′-O-methyl markers at the 3′-terminal^20^. Additionally, the presence of piRNAs in specific somatic tissues has been documented in *Drosophila* and primates^21–23^. With the rapid development of next generation sequencing technology, it is possible to precisely identify an increasing number of novel and weakly expressed small RNAs in non-model animals^24^.

Since the first piRNAs discovered in 2006, many piRNAs have been identified in organisms^15–19,22,25^. The primary function of piRNAs is believed to be silencing of transposable elements in the germline, although there is evidence that piRNAs have also been adapted for other functions, such as post-transcriptional gene silencing and functions in immune system^26^. Here, we identified and characterized putative piRNAs in somatic and gonadal tissues of pearl oysters by high-throughput sequencing. The discovery of abundant expression of piRNAs in a wide range of somatic tissues will increase understanding of the diversity and function of small RNAs in pearl oyster and closely related organisms. In particular, it will aid understanding of the physiological roles of small RNAs in mollusks, particularly in biomineralization.

## Results

### Sequencing of putative piRNAs from somatic and gonadal tissues of pearl oysters

**Figure 1:**
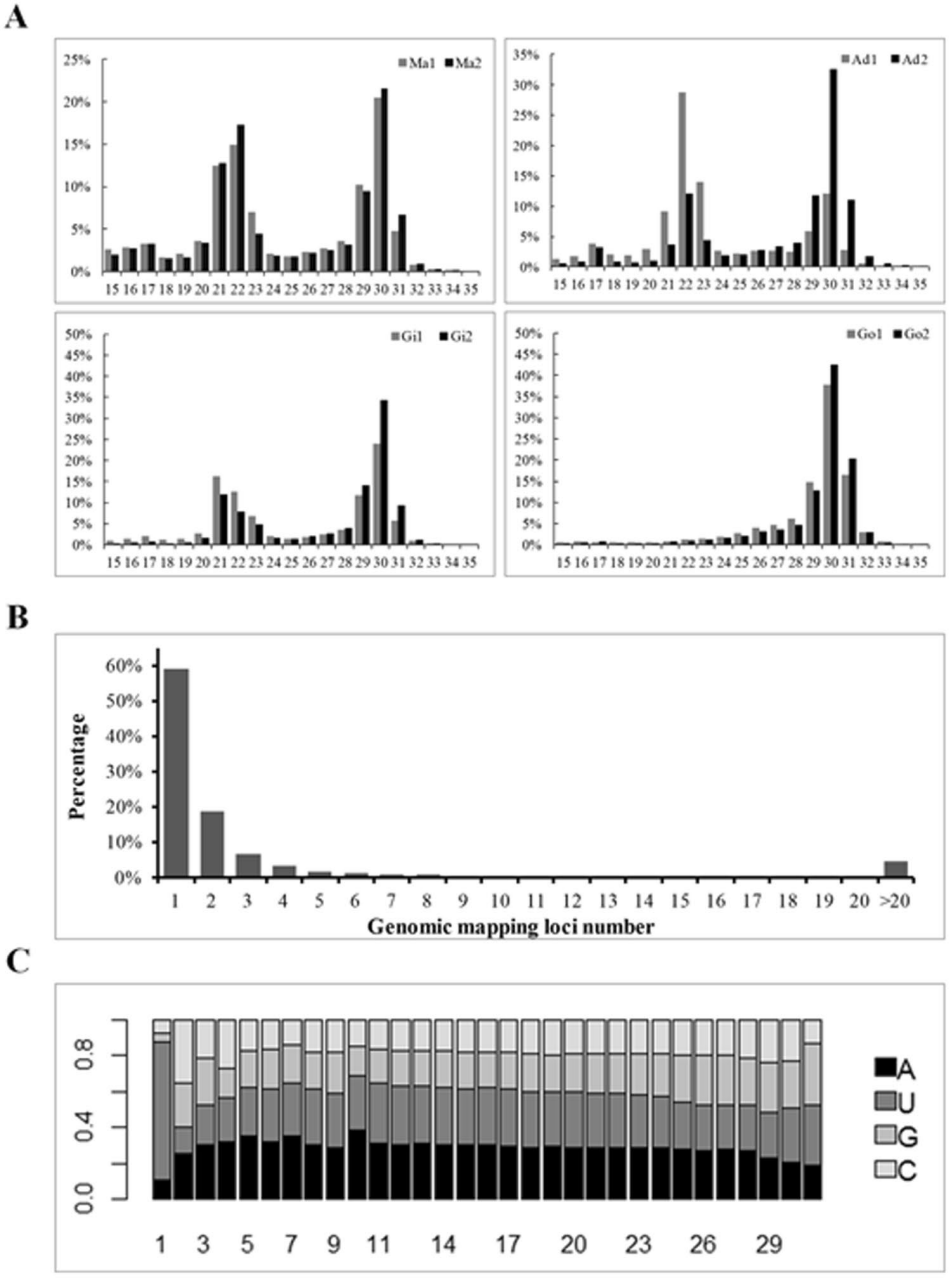
Putative piRNA sequence processing. (**A**). The size profile of small RNAs from the somatic and gonadal tissues of pearl oysters. The peak at 21–23 nt probably represents the microRNA and endogenous siRNA population. The peak at 26–31 nt probably represents putative piRNAs. Ma: mantle tissue; Ad: Adductor muscle; Gi: Gill tissue; Go: Gonad tissue. (**B**) Distribution of the number of loci at which each individual unique piRNA was mapped. A total of 0.82 million unique piRNAs with number of reads >1 were examined. (**C**) Base composition at each site of the unique piRNAs from the 5′ terminal.

Eight small-RNA libraries were sequenced using the Ion Proton system. We obtained 50.31 million raw reads. After removing the adapters, low-quality reads, reads with unknown nucleotides, and reads outside the 15–35 nucleotides range, 42.7 million reads (Table 1, Step 1) remained for length-distribution analysis (Fig. 1). Two obvious peaks (at 21–23 nt and 29–31 nt) were observed in the distribution of the lengths of small RNAs from somatic tissues, and one peak at 29–31 nt was found in small RNAs from the gonadal tissue (Fig. 1A). After removing known RNAs, including miRNAs, rRNAs, tRNAs, snRNAs, and snoRNAs, 31.5 million clean reads (Step 2) remained for analysis. The size profile of small RNAs after removed all the known RNAs were shown in Supplementary Fig. S1, which have a low percentage of 21–23 nt RNAs. Then 22.6 million reads were mapped to the pearl oyster reference genome (Step 3). Finally, 18.0 million reads were considered valid putative piRNA reads, ranging from 26 to 31 nt in length (Step 4).

**Table 1:** Summary of the number of reads at each processing step in the putative piRNA analysis.

Libraries	Ma1	Ad1	Gi1	Go1	Ma2	Ad2	Gi2	Go2	Total
Raw reads	5.57	1.96	13.59	6.74	8.54	5.14	5.74	3.04	50.3
Step 1	3.69	1.59	11.57	5.86	6.94	4.74	5.47	2.82	42.7
Step 2	2.37	0.80	7.46	5.71	4.45	3.84	4.15	2.74	31.5
Step 3	1.78	0.56	5.45	3.90	3.25	2.76	3.03	2.69	22.6
Step 4	1.20	0.33	4.19	3.37	2.23	2.24	2.69	1.70	18.0

Step 1: remove adapter sequence and the remaining reads of size 15–35 nt.

Step 2: remove known small RNAs.

Step 3: remove reads not mapped to the reference genome.

Step 4: select reads in the size range 26–31 nt.

Number of reads is in millions. Ma: Mantle tissues; Ad: Adductor muscle; Gi: Gill tissues; Go: Gonad tissue.

Consistent with the signature of piRNAs observed in previous studies, 59.31% unique putative piRNA reads were uniquely mapped to the reference genome and the remaining unique reads were mapped to multiple loci of the reference genome (Fig. 1B). Of the unique putative piRNAs, 70.1–87.8% began with a U in each sequencing library (Fig. 1C). This was also consistent with previously documented features of piRNAs. Therefore, we deduced that the 18.0 million reads were mostly derived from piRNAs, and we therefore used them for our subsequent analyses. The final dataset contained 2.8 million unique sequences. For simplicity, in the following sections we refer to the putative piRNA reads as “piRNAs”, and unique putative piRNA sequences as “unique piRNAs”.

**Figure 2:**
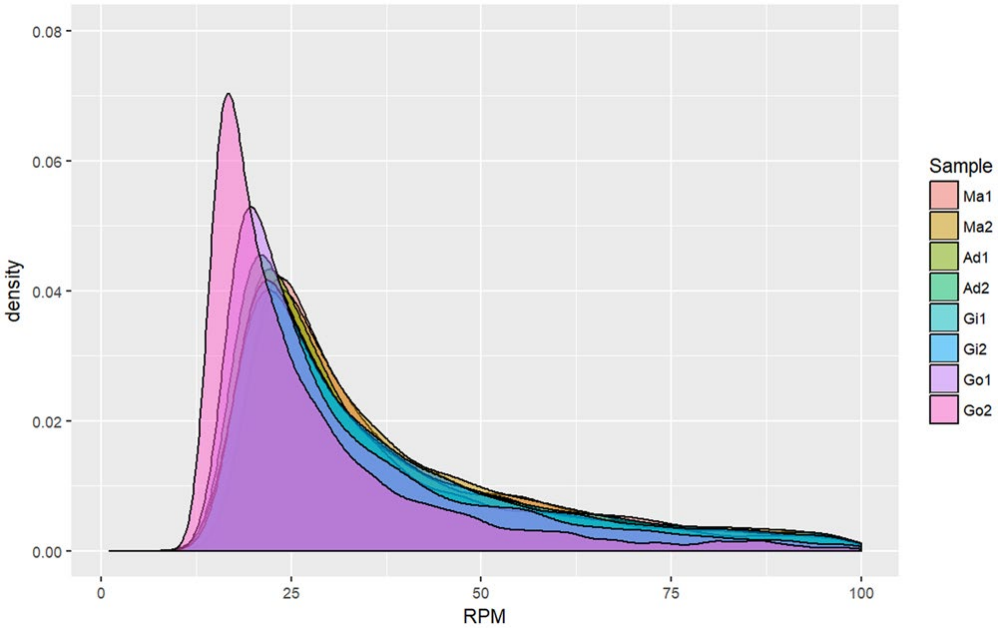
Expression density of putative piRNAs from the somatic and gonadal tissues of pearl oysters. (**A**) Mantle libraries from two pearl oysters. (**B**) Adductor muscle libraries from two pearl oysters. (**C**) Gill libraries from two pearl oysters. (**D**). Gonad libraries from two pearl oysters. Ma: Mantle tissues; Ad: adductor muscle; Gi: Gill tissue; Go: Gonad tissue.

Further examination of the piRNAs revealed that a piRNA (piRNA0001) species with sequence 5′-UACUUUAACAUGGCACAGAUAUAAUGACCU-3′, which was mapped on scaffold1502.1 with multiple tandem manners, had the highest number of reads per million (RPM) of the piRNAs expressed in the somatic tissues, and that piRNA 5′-ACAGAAAUCUCGGAUCCAUCUAUAGACAGG-3′ was the most strongly expressed in the gonadal tissues. The top 20 most-highly expressed piRNAs (7.7% of reads) in the two types of tissues are shown in Supplementary Table S1. piRNAs were highly expressed in both somatic and gonadal tissues (Fig. 2). Approximately 18,179–33,301 unique piRNAs had an RPM > 5. The gonadal tissues had more unique piRNA species with low RPMs than the somatic tissues did.

### Putative piRNA sequence mapping with the reference genome

It was thought that, in the organisms in which this has been studied, mature piRNAs originate from the processing of longer RNA precursors^27^. We mapped the putative piRNAs to the pearl oyster reference genome to identify piRNA gene clusters. The numbers of piRNA clusters identified based on different merge distances and criteria for minimum coverage are shown in Supplementary Fig. S2. We used moderate criteria (<1 kb merge distance and a minimum coverage of six reads) to identify piRNA clusters from 18.0 million piRNAs. A total of 35,848 piRNA clusters were defined, and the locations and reads per kilobase per million (RPKM) of each piRNA gene clusters are shown in Supplementary Table S2. Clusters are named in order in each scaffold. The sizes of the clusters ranged from 30 bp (single unique piRNA) to 60.9 kb, and 49.1% of the piRNA clusters were longer than 1 kb. piRNAs from 22,629, 24,933, 27,789, and 30,671 clusters were observed in the mantle, adductor muscle, gill, and gonad tissues, respectively. Putative piRNAs from 14,125 clusters were commonly expressed in all four tissues, whereas 2,896 clusters were exclusively observed in the gonadal tissues (Supplementary Fig. S3). A principal component analysis (PCA) of piRNA-cluster expression patterns from all eight libraries clustered those of the somatic tissues together and differentiated the somatic and gonadal tissues (Supplementary Fig. S4).

**Figure 3:**
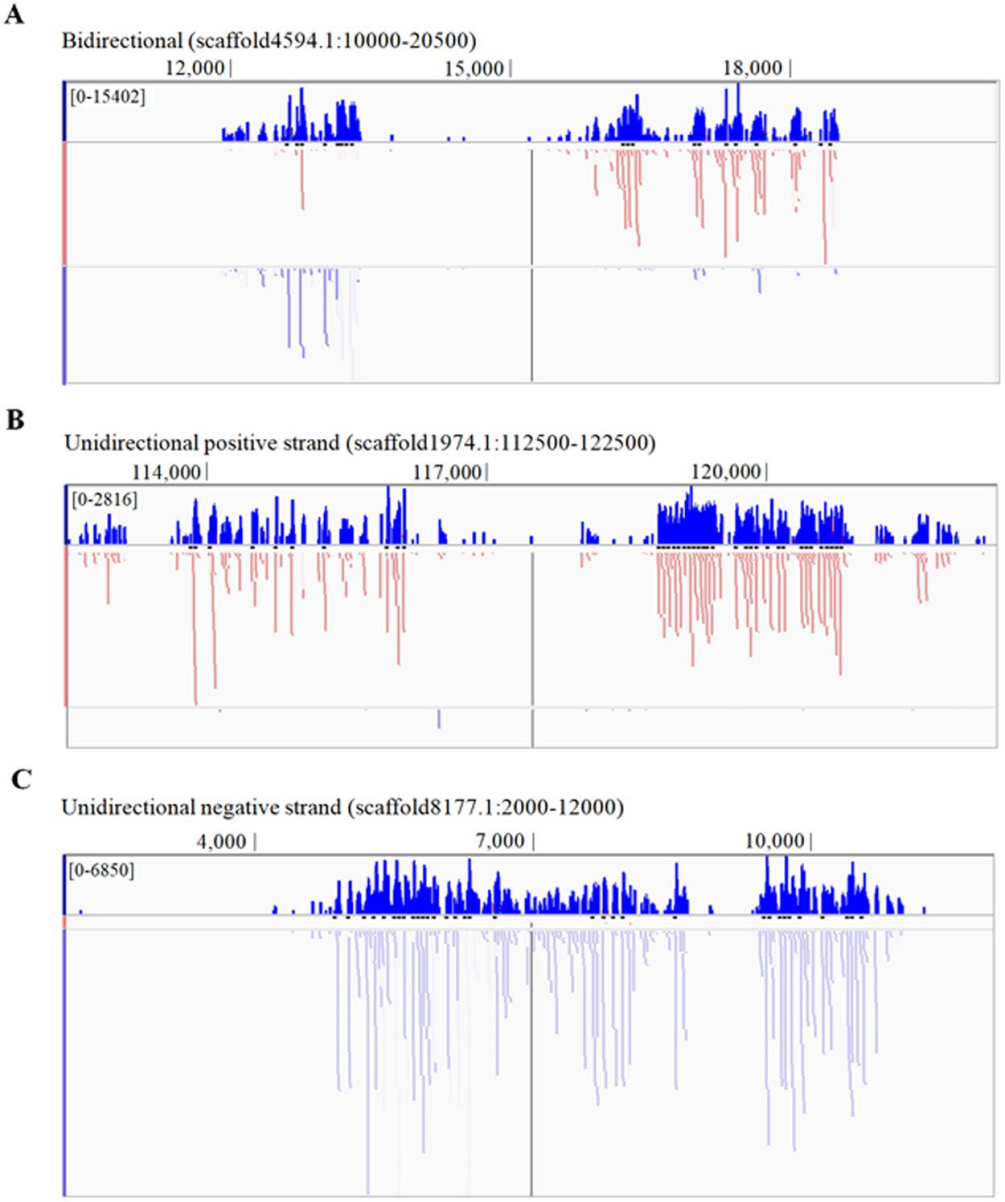
Results of mapping putative piRNAs to the reference genome. (**A**) Bidirectional mapping. (**B**,**C**) Unidirectional positive or negative strand mapping. The logs of piRNA mapping density (dark blue) and actual mapping for positive strand (light red) and negative strand (light blue) of the reference genome. The mapping density was plotted using the Integrative Genomics Viewer tracks tool.

**Figure 4:**
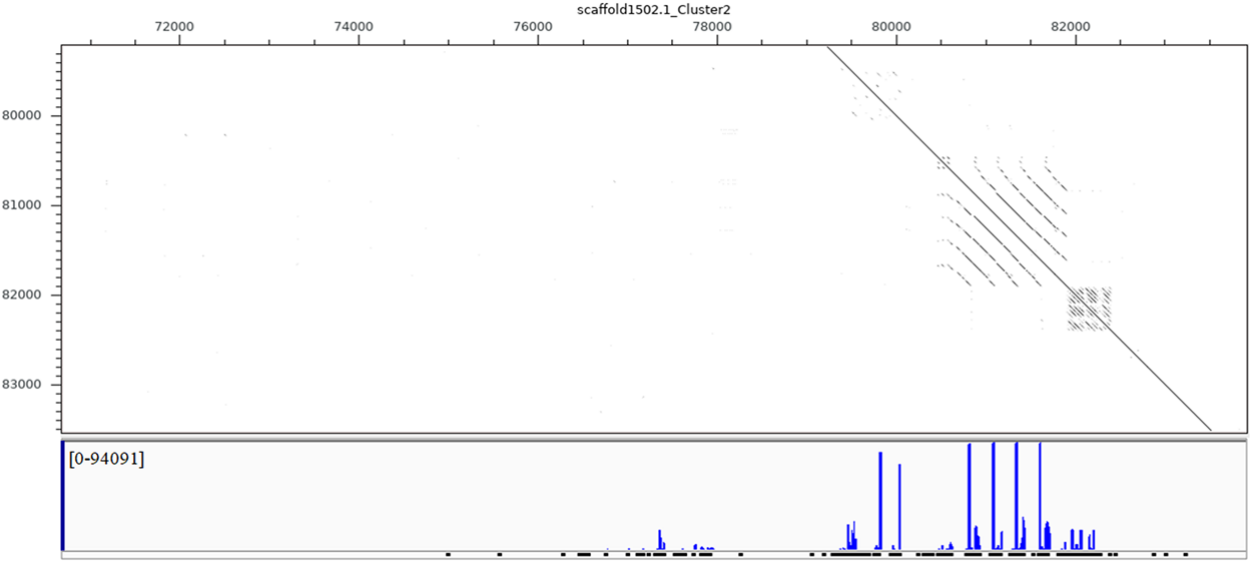
Example of putative piRNA mapping according to multiple tandem loci on the scaffold. The dotted lines illustrate homologous sequence, which represent repeats on the reference genome.

Mapping the piRNAs to the reference genome revealed two different mapping patterns: bidirectional and unidirectional (Fig. 3). We found that 40.69% of the unique piRNAs were mapped to the reference genome at multiple loci, and 25% of the unique piRNAs were mapped with multiple and tandem patterns on the same scaffold. Dot-matrix analysis of the piRNA gene clusters revealed that piRNAs were often encoded in a tandem manner (Fig. 4).

### Putative piRNA clusters expressed differently in somatic and gonadal tissues

Analysis of the expression of piRNA clusters aids understanding of their expression patterns in the somatic and gonadal tissues of the pearl oyster. PCA analysis indicated that piRNA clusters from the somatic tissues have similar expression patterns but differ from gonadal tissues. To investigate the difference between piRNA cluster patterns in the somatic and gonadal tissues, we conducted a differential expression analysis using edgeR, with the following criteria: FDR < 0.01, and an absolute log2Ratio value of >1. The results showed no difference in the expression patterns of piRNA clusters among the somatic tissues. However, 127, 30, and 377 piRNA clusters were significantly differently expressed in the mantle, adductor muscle, and gill tissues than in the gonadal tissues, respectively (Supplementary Table S3).

### β-elimination reaction confirms putative piRNA

**Figure 5:**
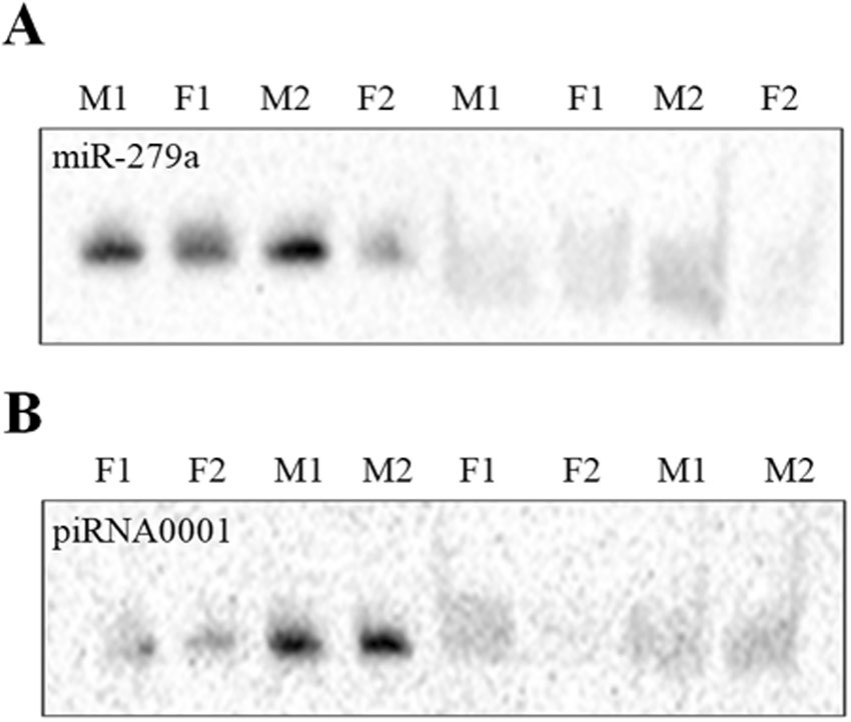
β-elimination reaction of miRNA and putative piRNA oligonucleotides. (**A**) In the absence of 2′-O-methylation at the 3′ terminal, treated miR-279a (right) showed increased migration relative to untreated miR-279a (left) in a 15% polyacrylamide gel. (**B**) piRNA0001 showed the same migration before and after treatment, because methylation at the 3′ terminal protected it from periodate oxidation. F1 and F2: Female individuals total RNA; M1 and M2: male individuals total RNA. Gel figures are cropped and toned. The original gel images are available in Supplementary Information.

piRNAs are modified by methylation at the 3′ terminal for protection from periodate oxidation. The β-elimination reaction identifies methylation at the 2′-OH of the 3′ terminal of small RNAs. The products of the β-elimination reaction of miR-279a showed increased migration in a 15% polyacrylamide gel, while the mobility of piRNA0001 was unaffected by the β-elimination reaction (Fig. 5), indicating that there is an important difference between miRNAs and putative piRNAs in terms of their molecular structure at the 2′-OH of the 3′ terminal.

### Detection of putative piRNA in somatic tissues

**Figure 6:**
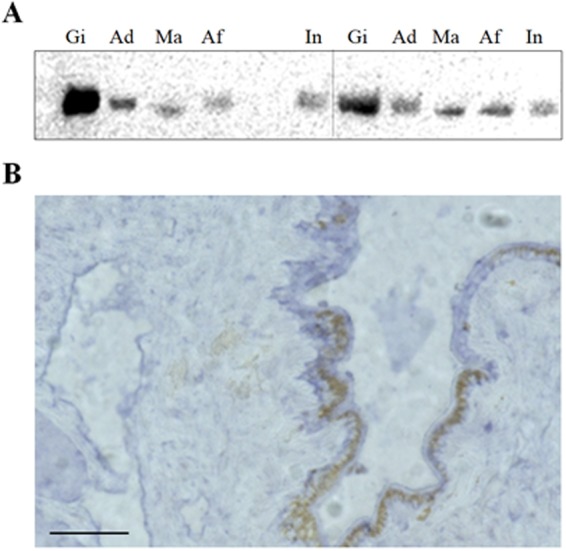
Detection of putative piRNAs in pearl oyster somatic tissues. (**A**) Northern blotting analysis of piRNA0001 expression in gill (Gi), adductor muscle (Ad), mantle (Ma), abdominal foot (Af), and intestine (In) tissues. (**B**) *In situ* hybridization analysis of piRNA0001 in mantle tissue. Bar = 50 μm. Gel figure is cropped and toned. The original gel image is available in Supplementary Information.

When RNA extracted from gill, adductor muscle, mantle, intestine, and abdominal foot tissues was used for piRNA detection by northern blotting and *in situ* hybridization, piRNA0001 was widely expressed in all of these tissues, particularly the gill tissue (Fig. 6A). The *in situ* hybridization of small RNAs also revealed the expression of piRNAs in mantle tissue (Fig. 6B).

## Discussions

In the present study, we examined the expression patterns of small RNAs in the mantle (Ma), adductor muscle (Ad), gill (Gi), and ovary (Go) tissues of pearl oysters. Our first experiment examined the expression patterns of miRNA species in pearl oysters. Surprisingly, we found that RNA species of approximately 30 nt were more abundant than those of approximately 22 nt, which is the length expected for miRNA and/or endogenous siRNA. The 30 nt RNA species were rich in uridine (U) residue at the 5′-end. In addition, β-elimination reactions confirmed the presence of 2′-O-methylation at the 3′ terminal of a representative 30-nt RNA. Since these are typical features of piRNA species, we regarded the 30 nt RNA species as putative piRNA in *P. fucata*.

After removing reads that were of poor quality or not within the target size range, two obvious peaks in the distribution of lengths of small RNAs were observed in somatic tissues, and dominant 29–31 nt small RNAs observed in gonadal tissues. Four steps of sequence processing identified 18.0 million putative piRNAs and 2.8 million unique piRNAs. Abundant putative piRNAs were identified and characterized in pearl oyster somatic and gonadal tissues. Northern blotting and *in situ* hybridization revealed widespread expression of these RNA species in pearl oyster somatic tissues. Mapping of the putative piRNAs to the reference genome of the pearl oyster revealed that they were encoded in multiple loci, with tandem patterns on the genome. Also, their expression patterns were notably different in somatic and gonadal tissues. These results provide useful information for further research on piRNA in pearl oysters, and on the functions and molecular regulatory mechanisms of small RNAs in mollusks.

The main characteristics of most of the putative piRNAs—that they started with uridine at the 5′ terminal and were within the range 26–31 nt—are consistent with piRNAs in other animals^28–30^. A common feature of piRNAs in all systems analyzed thus far is that they derive from repetitive transposon rich regions of the genome^20,31^. In the piRNA biogenesis pathway, long piRNA precursors are transcribed from specific genomic loci known as piRNA clusters, cleaved and modified in the cytoplasm, and then transported into the nucleus in a complex with Piwi protein^32^. Due to the limited database of transposable elements in the pearl oyster, we performed a piRNA cluster analysis: we merged piRNA mapping loci within a distance of <1 kb and with a minimum coverage of six reads. A total of 35,848 piRNA clusters were identified on the reference genome, varying in length from 30 bp (single unique piRNA) to 60.9 kb, similar to piRNA clusters that have been identified in vertebrates and other invertebrates^29,33^. Twenty-five percent of the unique piRNAs mapped to multiple tandem loci on the scaffold, and comparisons between the dot-matrix analyses of piRNA clusters and piRNA genomic loci showed that most piRNAs were located on repeats on the scaffold. Previous report indicated that 54–65% of the piRNAs were mainly generated from repeat regions^22^. The result indicated that the biogenesis of piRNA in *P. fucata* is essentially common with those of other reported species. Our low repeat-mapping percentage may be the result of the imperfect pearl oyster reference genome, with 29,306 scaffolds^4^. Another important biogenesis region of piRNAs is the 3′-UTR regions^34,35^, but the relationship between piRNAs and the exonic regions of genes was beyond the scope of this study.

To gain better understanding of the putative piRNA expression pattern in the somatic and gonadal tissues, the RPKM of each piRNA cluster was used to compare piRNA expression among tissues. We found that 39.4% of piRNA clusters were commonly expressed in all tissues, and that 49.4% occurred in all somatic tissues. The PCA analysis of piRNA cluster density clustered the somatic tissues’ libraries together, indicating that overall, the piRNA clusters expressed in somatic tissues were not substantially different. Moreover, piRNA clusters were expressed differently in the mantle, adductor muscle, and gill tissues, respectively, from the gonadal tissue. Thus expression patterns in the pearl oyster are different in the somatic and gonadal tissues.

The piRNAs tested from *Drosophila*, zebrafish, and mouse are resistant to periodate treatment, indicating the occurrence of a modified 2′-OH at the 3′ terminal^36^. This 2′-O-methylation at the 3′ terminal is recognized as a defining feature of piRNAs in all animals studied to date^37–39^. Consistent with these findings, a β-elimination reaction confirmed the presence of a 2′-OH modification at the piRNA 3′ terminal. Thus, it is likely that piRNAs from pearl oysters carry a 2′-O-methylation at their 3′ terminal and display similar characteristic features to the piRNAs of mammals and flies^20,40^. Lastly, northern blotting and *in situ* hybridization further verified the expression of piRNAs in the somatic tissues of pearl oysters.

In this study, millions of putative piRNAs revealed a fascinating and unanticipated dimension of pearl oyster biology. The piRNA pathway is commonly thought to be germline specific, even though the somatic function of Piwi proteins was documented when they were first discovered^41,42^. However, new studies are currently re-exploring the Piwi–piRNA pathway in the somatic cells of a diverse range of organisms, and notable insight has recently been gained into the somatic function of the pathway in the ovarian somatic cells of *Drosophila*^21^. In addition, groundbreaking work in the lower eukaryotes has demonstrated a conserved function for Piwis and piRNAs in the somatic tissues, particularly in stem cells^32,43,44^. Piwi genes are expressed in the planarian totipotent stem cells that repopulated all somatic and germline lineages in planarians^45^. Furthermore, recent studies suggest that piRNAs are also associated with neuronal Piwi proteins and contribute to neuronal development and function^46,47^. Thus, it is evident that the diversity of functions of piRNAs is greater than previously thought. In the present study, putative piRNAs partially generated from repeats shared characteristic features with piRNAs identified in other organisms, and were strongly expressed in somatic and gonadal tissues, although with different expression patterns. However, the roles of piRNAs in the pearl oyster have remained unknown. They may play important roles beyond those of the germline in mollusks. Further studies are needed to determine whether piRNAs play a critical role in epigenetic silencing through DNA/chromatin methylation, similar to their germline homologs in mollusks^48^.

## Methods

### Ethics

This study was conducted in strict accordance with the recommendations in the University of Tokyo’s Guide for the Care and Use of Laboratory Animals. All efforts were made to minimize animal suffering.

### Experimental samples and tissue collections

Two female pearl oysters (approximately two years of age, with body length and body weight of 13 ± 2 cm and 95 ± 10 g, respectively) were collected from a pearl oyster culture population and cultured at the Mikimoto Pearl Research Institution, Mie Prefecture, Japan. The pearl oysters were put in ice water for anesthetization and then dissected for tissue collection. Mantle, adductor muscle, gill and ovary tissues were collected and stored in 2 ml tubes for later separation of the RNA (Thermo Fisher, Japan). The samples were stored overnight at 4 °C, and then preserved at −80 °C until use.

### Small-RNA extraction, library construction, and sequencing

Eight total RNA samples were extracted from the pearl oyster somatic and gonadal tissues. The subsequent small RNA extraction conformed to the protocol for the mirVana miRNA Isolation Kit (Life Technologies, America). Briefly, the sequencing libraries were constructed as per the Ion Total RNA-seq Kit v2 small-RNA library construction protocol (Life Technologies, America). Adapter ligation, synthesis of cDNA by reverse transcription, purification of the small-RNA fraction, and amplification by PCR were then performed to construct the cDNA libraries. Agilent 2200 Bioanalyzer (Agilent Technologies, Germany) was used for cDNA concentration checking. Using these cDNA libraries, a template for loading onto the analysis chip was prepared according to the protocol of the Ion PI Template OT 2200 Kit v3 (Life Technologies, America). Sequencing was then performed according to the protocol for the Ion Proton system (Ion Proton Sequencer and Ion Proton Torrent Server, Life Technologies, America).

### Putatvie piRNA sequence processing

The raw sequencing data were saved as FASTQ files. The PRINSEQ program was used to exclude data with an average quality score of ≤20, and reads with lengths outside the 15–35-nt range were removed from FASTQ files (Table 1, Step 1). Then length distribution of the filtered reads was analyzed and illustrated. Reads were then blasted against pearl oyster miRNAs (from our unpublished work), tRNA (from the *P. fucata* reference genome sequence by tRNAscan-SE^49^), the NCBI non-coding RNA database (http://blast.ncbi.nlm.nih.gov/), the Rfam database (ftp://sanger.ac.uk/pub/databases/Rfam/), and pearl oyster ribosome RNA (28s rRNA: AB214477.1, 18s rRNA: AB214463.1 and 5.8s rRNA: AB205102.1), to separate out miRNA, tRNA, snRNA, snoRNA, and rRNA sequences (Table 1, Step 2). We then mapped the remaining reads to the *P. fucata* reference genome (Version 2, http://marinegenomics.oist.jp/pearl/viewer/info?project_id=36) using Bowtie (version 1.2.1), allowing up to one mismatch and multiple matches (-v1; Table 1, Step 3). Reads which were within the size range 26–31 nt and mapped to the assembled scaffolds were selected as putative piRNAs (Table 1, Step 4).

The base biases of each site of the unique piRNAs were analyzed using R software (Version 3.3.2). The reads per million (RPM) value $[{\rm{RPM}}=(\frac{\,\mathrm{Number}\,{\rm{of}}\,{\rm{a}}\,{\rm{unique}}\,{\rm{piRNA}}\,{\rm{reads}}}{{\rm{Total}}\,{\rm{number}}\,{\rm{of}}\,{\rm{piRNA}}\,{\rm{reads}}\,{\rm{from}}\,{\rm{given}}\,{\rm{library}}})\times {10}^{6}]$ was used to compare the expression of unique piRNAs between libraries. The density of unique piRNAs in each library was investigated.

### Normalized putative piRNA cluster identification

piRNA clusters were identified from putative piRNAs using the following procedure: Firstly, all piRNA reads were mapped to the reference genome using “bwa mem”. The piRNA genomic mapping loci were merged, and we defined a region with a distance of <1 kb between any piRNAs as a merged region (bedtools merge –d 1000). Total reads were then re-mapped to the merged region to calculate the coverage (bedtools coverage –a –b). Here, we defined merged regions with a minimum coverage of six reads as a piRNA cluster, and counted the normalized number of piRNAs in each cluster using the measure reads per kilobase per million mapped reads (RPKM)^50^. The normalized piRNA enrichment in each cluster was defined as follows:

$$\frac{{\rm{Number}}\,{\rm{of}}\,{\rm{reads}}\,{\rm{mapped}}\,{\rm{to}}\,{\rm{a}}\,{\rm{piRNA}}\,{\rm{cluster}}\times {10}^{6}}{{\rm{Total}}\,{\rm{number}}\,{\rm{of}}\,{\rm{piRNA}}\,{\rm{reads}}\,{\rm{from}}\,{\rm{given}}\,{\rm{library}}\times {\rm{piRNA}}\,{\rm{cluster}}\,{\rm{length}}\,{\rm{in}}\,{\rm{kb}}}$$

Our definition of RPKM was slightly different from the conventional one, since piRNA precursor transcript information was not available, and our RPKM was normalized by genomic DNA length rather than transcript length. Finally, Dotter^51^ and the Integrative Genomics Viewer (IGV) tracks tool^52^, respectively, were used to visualize the repeats and the mapping density on the scaffold.

### Differential expression of putative piRNA clusters in the somatic and gonadal tissues

The mapped reads were normalized by cluster length according to RPKM for each piRNA cluster in the somatic and gonadal tissues, which facilitated comparisons of piRNA levels between tissues. The edgeR package^53^ was used to identify differently expressed piRNA clusters between two tissues. Clusters that were differently expressed in two tissues were identified using the following filter delimitations: FDR (false discovery rate) <0.01; and absolute value of log2Ratio > 1.

### β-elimination reaction of miRNA and putative piRNA

Previous studies have shown that the homolog gene of *HEN1* mediates piRNA 2′-O-methylation at the 3′ terminal^54,55^. The β-elimination reaction was used to identify methylation at the 3′ terminal of small RNAs. The detailed process of the β-elimination reaction has been described previously^56^. Briefly, an NaIO_4_ solution was used to cleave the vicinal hydroxyls to a dialdehyde, and then the dialdehyde was treated with borate buffer at pH 9.5 (β-elimination reaction) in the dark at room temperature for 30 min. This resulted in a 3′-monophosphate of the miRNA that was shorter by 1 nt, which had a higher migration rate in gel electrophoresis than the miRNA did prior to the treatment^57^. Although the β-elimination reaction also resulted in methylation at the 2′-OH of the 3′ terminal of the piRNAs, these methylated piRNAs had the same migration rate in gel electrophoresis. The β-elimination reaction was used to investigate the modification of the 3′ terminal of small RNAs. We confirmed the most strongly expressed piRNA (piRNA0001: 5′-UACUUUAACAUGGCACAGAUAUAAUGACCU-3′) displayed 3′ terminal methylation, and we used miR-279a as a control. Four total RNAs from *P. fucata* gill tissues were used for small RNA β-elimination reaction analysis. To increase binding specificity and affinity to the targets, locked nucleic acid (LNA) probes were designed and used to detect small RNAs by northern blotting. Both terminals of the probes were labeled with digoxigenin. The process of northern blotting was conducted as previously described^58,59^, and the northern blotting probes are shown in Table 2.

**Table 2:** Antisense probes for the detection of miRNAs and putative piRNAs.

Small RNA	Sequence	DNA/LNA (capitalized) probe sequence
miR-279a	5′-UGACUAGAUCCACACUCAUCCU-3′	5′-GgaTgaGtgTggAtcTagTca-3′
piRNA0001	5′-UACUUUAACAUGGCACAGAUAUAAUGACCU-3′	5′-GcaAgtAgtTgaCgtAgtCc-3′

### Detection of putative piRNA in somatic tissues

The most strongly expressed putative piRNA (piRNA0001) in somatic tissues was selected for detection analysis in pearl oyster. Six pearl oysters (three of each sex) were collected from Mie Prefecture, Japan. After anesthesia with ice water, five somatic tissues (gill, mantle, adductor muscle, intestine, and abdominal foot) were dissected for sampling. The process of total RNA extraction was as described above. When sampling, part of the mantle tissue was stored in 4% paraformaldehyde at 4 °C for 12 hours, which was then replaced with 10%, 15%, and 20% sucrose for long-term preservation. We conducted the *in situ* hybridization of small RNAs using the IsHyb *In Situ* Hybridization Kit protocol (Biochain, America).

### Accession codes

All sequencing data are deposited in the DNA Data Bank of Japan (DDBJ) 491 database under accession DRA006953.

## Electronic supplementary material

Supplementary information

Dataset1

Dataset2

Dataset3
