# Supplementary material for: Piwi-interacting RNA (piRNA) expression patterns in pearl oyster (Pinctada fucata) somatic tissues
Source: Sci Rep. 2019 Jan 22;9:247. doi: 10.1038/s41598-018-36726-0 (PMC6342924; doi:10.1038/s41598-018-36726-0)
Supplement: Supplementary file 1 — Supplementary information [file 41598_2018_36726_MOESM1_ESM.docx]

**Supplementary information:**

Piwi-interacting RNA (piRNA) expression patterns in pearl oyster (*Pinctada fucata*) somatic tissues

Songqian Huang^1, *^, Yuki Ichikawa^1, *^, Yoji Igarashi^1^, Kazutoshi Yoshitake^1^, Shigeharu Kinoshita^1^, Fumito Omori^2^, Kaoru Maeyama^2^, Kiyohito Nagai^3^, Shugo Watabe^4^, Shuichi Asakawa^1^

^1^Graduate School of Agricultural and Life Sciences, The University of Tokyo, Bunkyo-ku, Tokyo 113-8657, Japan. ^2^Mikimoto Pharmaceutical CO., LTD., Kurose 1425, Ise, Mie 516-8581, Japan. ^3^Pearl Research Laboratory, K. MIKIMOTO & CO., LTD., Osaki Hazako 923, Hamajima, Shima, Mie 517-0403, Japan. ^4^School of Marine Biosciences, Kitasato University, Minami-ku, Sagamihara, Kanagawa 252-0313, Japan. ^*^These authors contributed equally to this work. Correspondence and requests for materials should be addressed to S.A. (email: asakawa@mail.ecc.u-tokyo.ac.jp)

**Supplementary Figure S1.** The size profile of small RNAs from the somatic and gonadal tissues of pearl oysters after removed all the known RNAs. The peak at 21–23 nt probably represents the microRNA and endogenous siRNA population. The peak at 26–31 nt probably represents putative piRNAs.


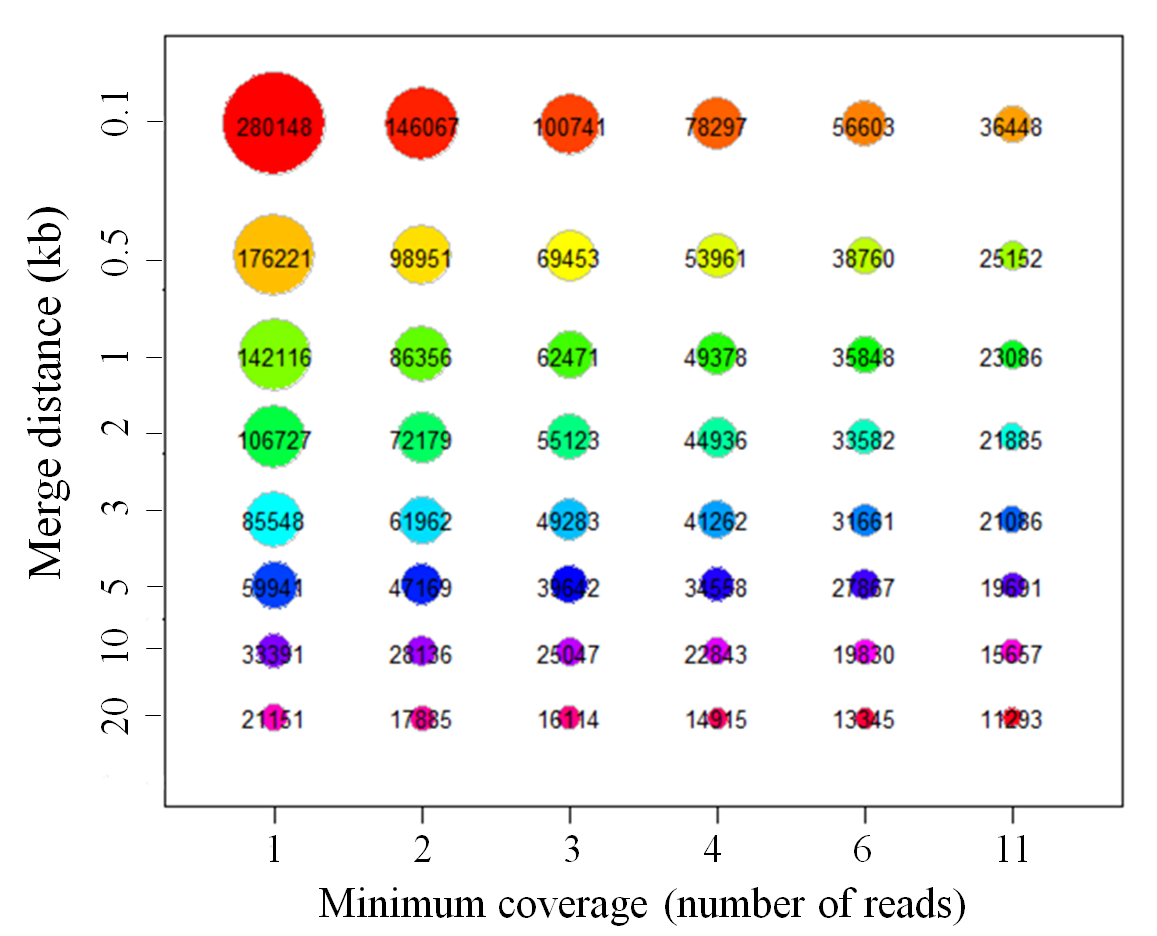


**Supplementary Figure S2. Numbers of putative piRNA clusters obtained using different merge distances and criteria for minimum coverage.** The number in each circle is the number of piRNA clusters obtained for that merge distance and minimum coverage criterion.

**
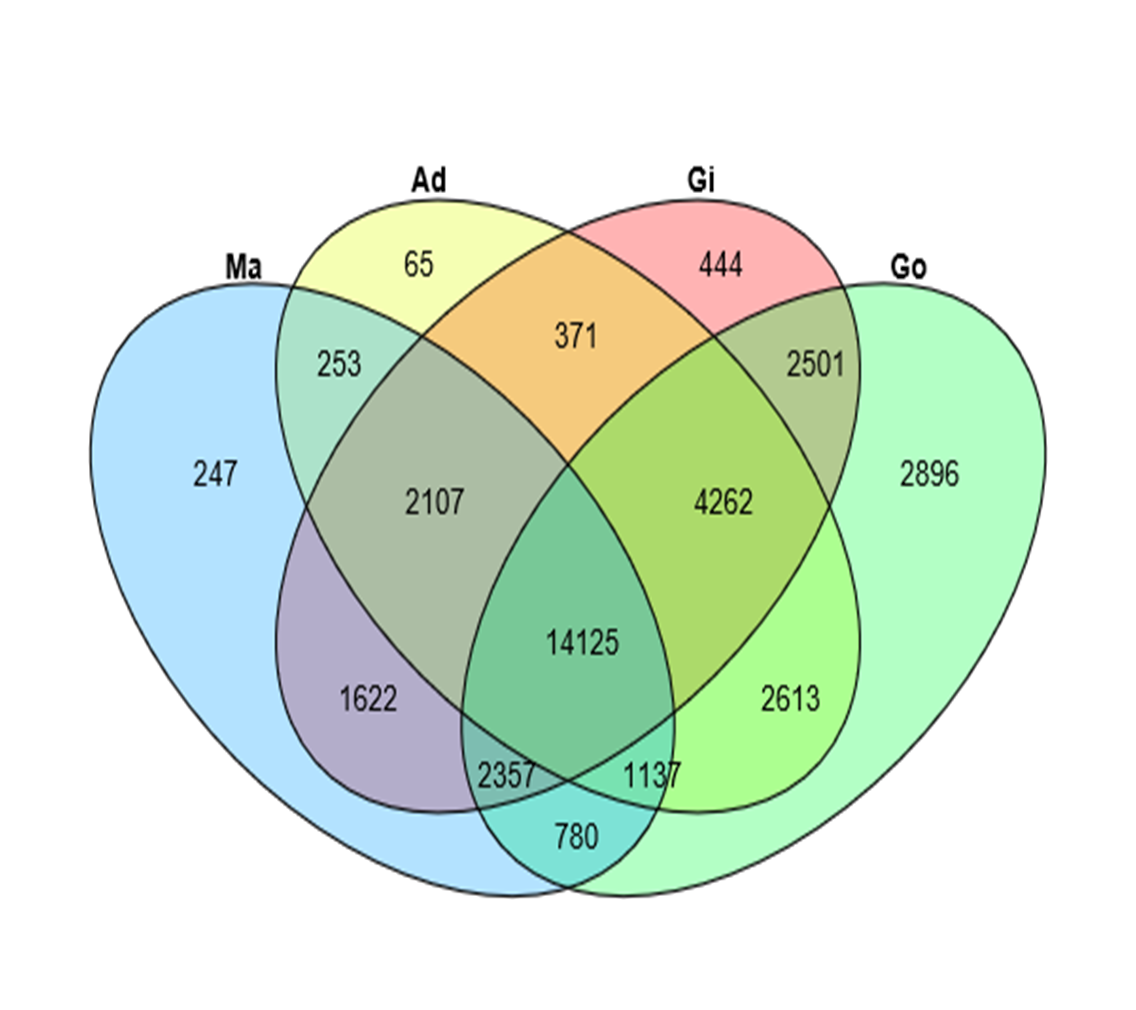
**

**Supplementary Figure S3. Number of putative piRNA clusters observed in the somatic and gonadal tissues of the pearl oyster.** 89.81% piRNA clusters were observed in more than one of the somatic tissues, while 2,896 (8.08% of total piRNA clusters) were expressed exclusively in the gonadal tissues. Ma: Mantle tissue; Ad: Adductor muscle; Gi: Gill tissue; Go: Gonad.


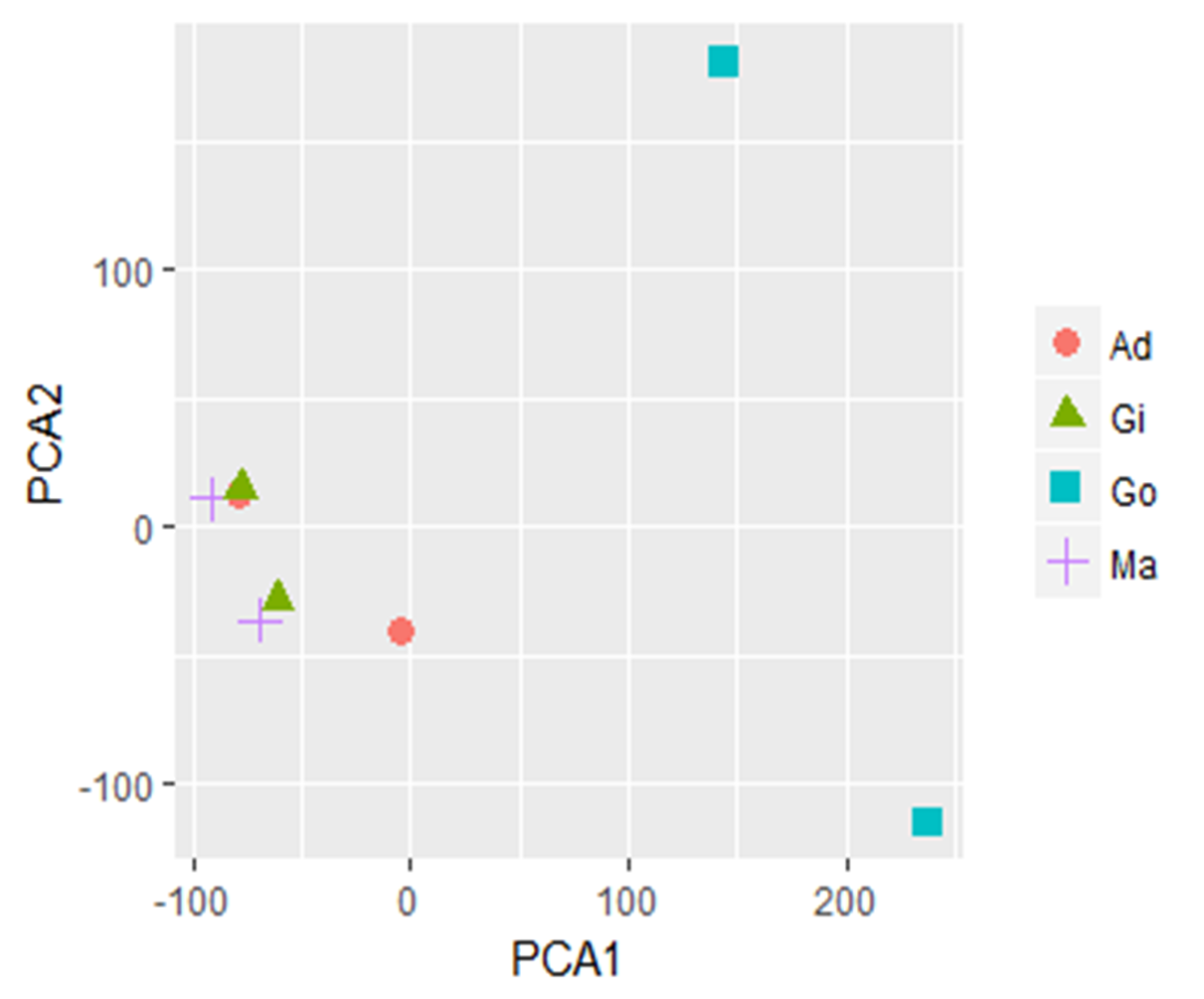


**Supplementary Figure S4.** **PCA analysis of the expression patterns of putative piRNA clusters in the somatic and gonadal tissues of the pearl oyster.** piRNA expression patterns of somatic tissues cluster together, and far from gonadal tissue, which reveal that two different expression patterns in somatic and gonadal tissues. Furthermore, two dissimilar Go piRNA expression patterns show variability in gonandal tissues in *P. fucata*. Ma: Mantle tissue; Ad: Adductor muscle; Gi: Gill tissue; Go: Gonad.


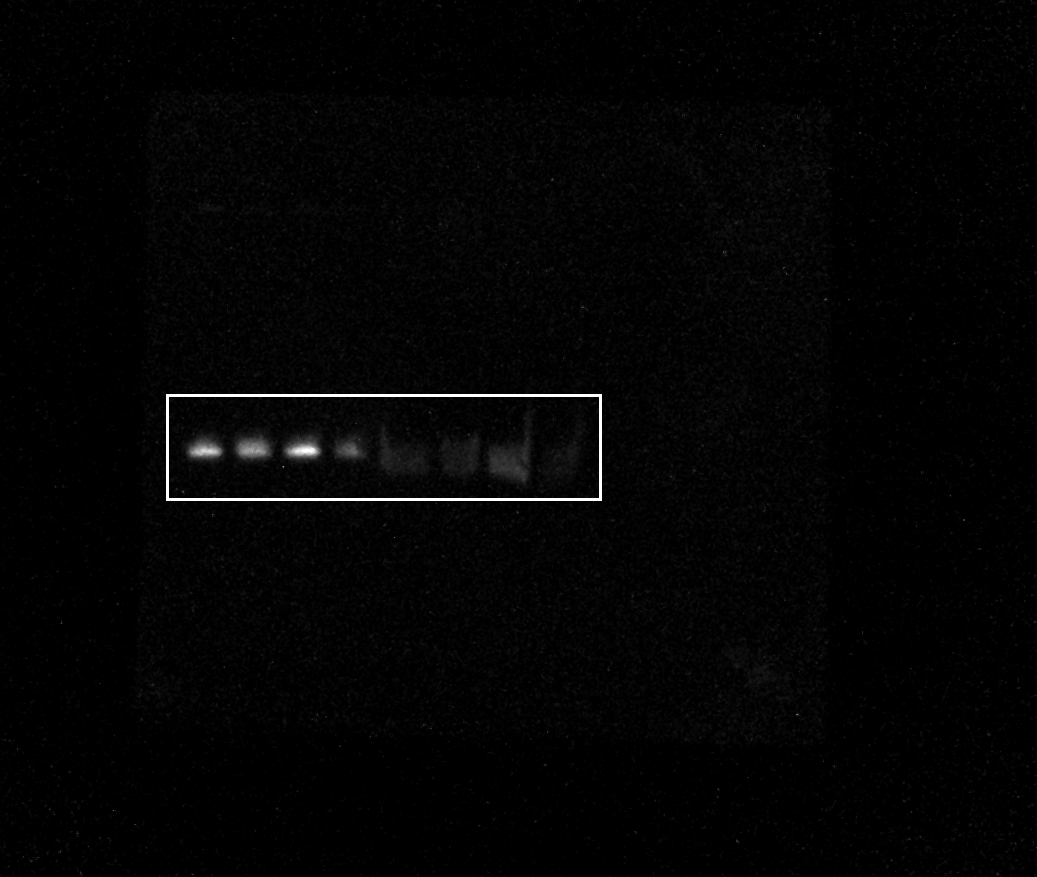


**Supplementary Figure S5.Original gel image. The white border is cropped and toned for Figure 6A.**


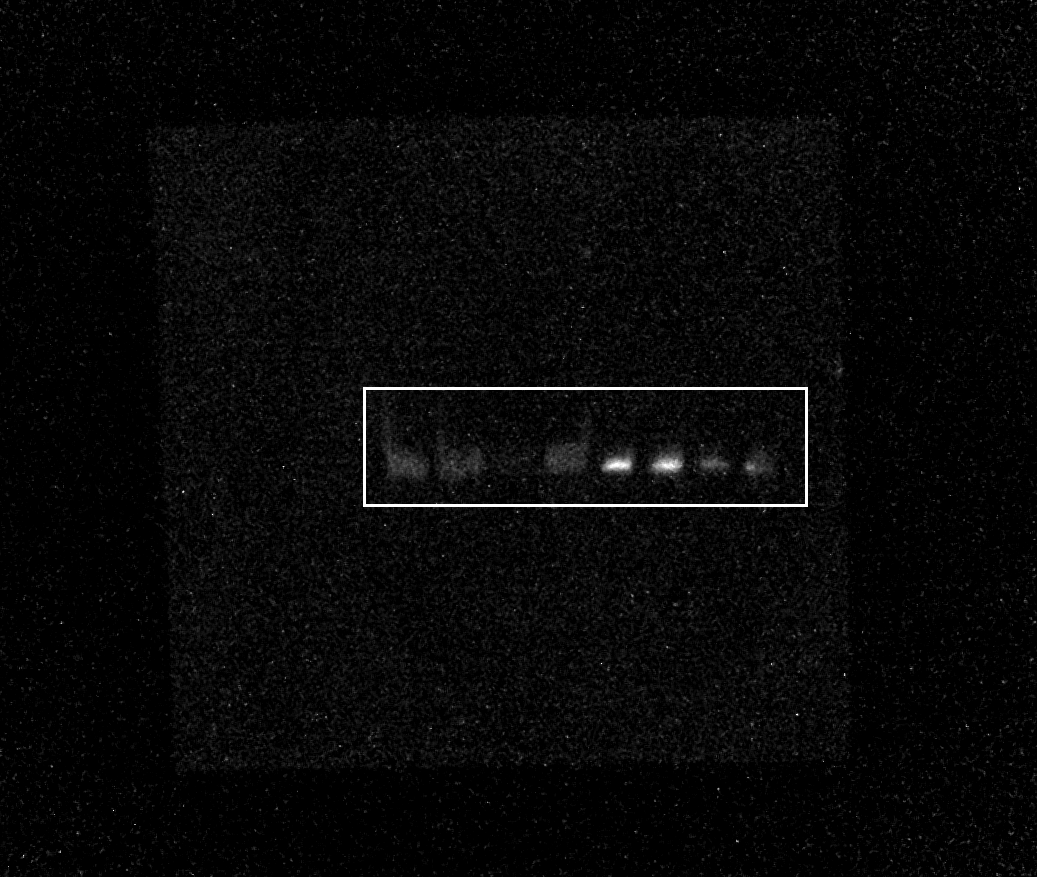


**Supplementary Figure S6.Original gel image. The white border is cropped, toned and flipped horizontally for Figure 6B.**


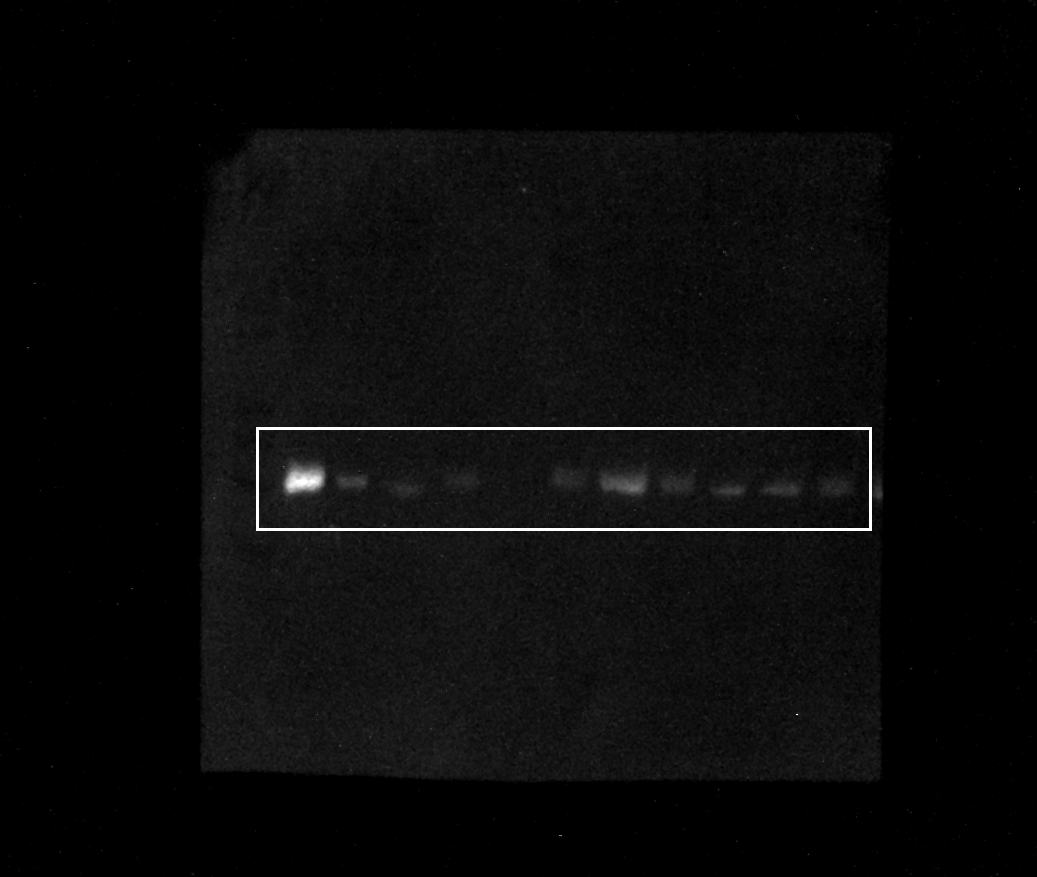


**Supplementary Figure S7.Original gel image. The white border is cropped and toned for Figure 7A.**

**Supplementary Dataset File:**

Table S1 The top 20 most-strongly expressed putative piRNAs in the somatic and gonadal tissues of the pearl oyster.

Table S2 Location and reads per kilobase per million (RPKM) of each piRNA cluster from somatic and gonadal tissues of the pearl oyster.

Table S3 Putative piRNA clusters that were expressed differently in somatic and gonadal tissues of the pearl oyster.
